# Self-reported periodontitis and C-reactive protein in Parkinson’s disease: a cross-sectional study of two American cohorts

**DOI:** 10.1038/s41531-022-00302-1

**Published:** 2022-04-13

**Authors:** Patrícia Lyra, João Botelho, Vanessa Machado, Silvia Rota, Ryan Walker, Juliet Staunton, Luís Proença, Kallol Ray Chaudhuri, José João Mendes

**Affiliations:** 1 Clinical Research Unit (CRU), Centro de Investigação Interdisciplinar Egas Moniz (CiiEM), Egas Moniz—Cooperativa de Ensino Superior, Almada, Portugal; 2 Evidence-Based Hub, CRU, CiiEM, Egas Moniz—Cooperativa de Ensino Superior, Almada, Portugal; 3 King’s College London, Department of Basic & Clinical Neuroscience, Institute of Psychiatry, Psychology & Neuroscience, King’s College London, London, UK; 4 Parkinson’s Foundation Center of Excellence, King’s College Hospital, London, UK; 5 King’s College Hospital NHS Foundation Trust, London, UK; 6 Quantitative Methods for Health Research (MQIS), CiiEM, Egas Moniz—Cooperativa de Ensino Superior, Almada, Portugal

**Keywords:** Parkinson's disease, Parkinson's disease, Epidemiology

## Abstract

Periodontitis triggers systemic repercussions, such as elevated levels of high-sensitive C-reactive protein (hs-CRP). This has never been studied within Parkinson’s Disease (PD). The aim of this study is to compare hs-CRP levels of self-reported periodontitis cases versus cases without periodontitis in PD patients. Data from the National Health and Nutrition Examination Survey (2015–2016 and 2017–2018 waves) were analyzed. PD cases were identified through medication regimens and periodontitis cases through a validated self-report questionnaire. 51 participants were included (24 females, 27 males, with mean age of 62.96 (14.71)). While the self-reported periodontitis group presented elevated levels of circulating hs-CRP (5.36 vs. 1.99 mg/L, *p* = 0.031), the self-reported without periodontitis group presented higher lymphocyte levels (29.35 vs. 28.03%, *p* = 0.007). Blood levels of hs-CRP were significantly higher in PD cases with self-reported periodontitis. Apart from the lymphocyte levels, there were no other significant differences according to the self-reported periodontal status. Future studies shall explore this association using clinical measures.

## Introduction

Parkinson’s Disease (PD) is the fastest growing neurodegenerative movement disorder, affecting around 10 million people worldwide^1,2^. This chronic, progressive and degenerative condition of both the peripheral and central nervous systems^3,4^ is clinically heterogeneous, with various motor and non-motor clinical features^5^. It has been hypothesized that the onset and progression of PD, unclear thus far, is dependent on the conjugation of different key factors such as neuroinflammation, alpha-synuclein induced neuronal dysfunction (through intracellular aggregation into Lewy bodies, which stand as the pathological hallmark of PD), systemic chronic inflammation (translated in the dysregulation of circulating inflammatory cytokines) and even gut and periodontal dysbiosis^6,7^.

In the advanced stages, beyond the debilitating and interfering impact of motor and non-motor symptoms (NMS) on everyday-life activities, PD also has a major detrimental effect on patients’ overall quality of life^5,8^. Oral health is no exception and may be deteriorated in PD resulting from impaired oral hygiene and lack of oral care^9–17^.

Among the possible oral conditions that may arise from inadequate oral care is periodontitis, a chronic, infectious, and inflammatory condition characterized by the destruction of the periodontium^18^. The physiopathology of periodontitis involves dental plaque dysbiosis and an uncontrolled immune response attacking the periodontal tissues^19^. Even though a clinical periodontal diagnosis is a gold standard, the self-report of periodontitis is an interesting epidemiological strategy that has been successfully developed and validated^20–22^. As an example, a recent prospective cohort study analyzed self-reported periodontitis relationship with female fecundability^23^, showing the potential of this self-reported measure in epidemiological scenarios.

The mutual link between PD and periodontitis has been studied recently. On the one hand, fine motor impairments and cognitive decline in PD patients compromise oral hygiene habits and general oral health status^9–16,24^. On the other hand, evidence has surged on bacterial inflammagens—including major virulence factors of key periodontal pathogens such as Porphyromonas gingivalis, like lipopolysaccharide (LPS) and gingipains—fueling a systemic inflammatory state that might be involved on the development of PD^4,25^. Furthermore, periodontitis was associated with a leukocytosis state in PD patients^26^. Also, higher blood levels of amyloid beta were found in periodontitis, mediated by inflammatory markers such as IL-6 and high-sensitive C-reactive protein (CRP)^27^. In fact, CRP is a widely evaluated non-specific biomarker in the clinical context, not only in the diagnosis and monitoring of acute inflammatory and infectious events but also in the management and prediction of chronic inflammatory conditions, such as cardiovascular and neurodegenerative diseases^28,29^. There are also increased levels of pro-inflammatory cytokines in PD, including CRP^6^. However, hs-CRP levels have never been studied in PD cases according to their periodontal status, and this may provide useful information in the PD-periodontitis link regarding its systemic inflammatory burden.

Hence, we aimed to compare the hs-CRP levels of individuals with PD, according to their self-reported periodontal status.

## Results

### Population

**Fig. 1:**
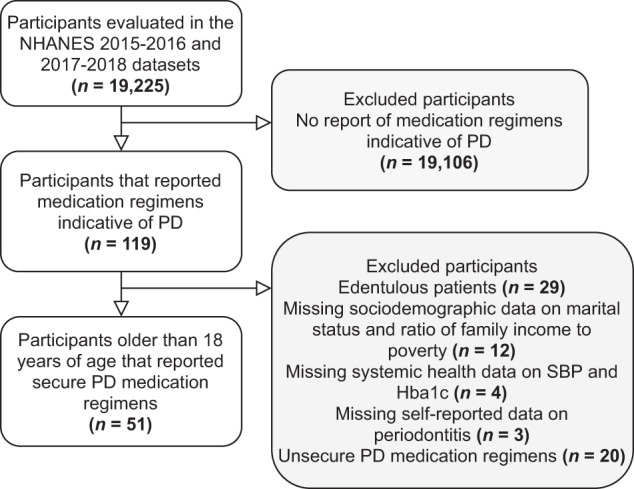
Flowchart of patient inclusion. Data describes the steps involved in the inclusion of the final sample with the number of patients and respective reasons.

From the 19,225 evaluated participants of the 2015–2016 and 2017–2018 NHANES waves, 119 reported medication regimens indicative of PD, and 51 adults were included for analysis with the detailed reasons for exclusion presented in the flowchart of Fig. 1.

The sample consisted of 24 females (47.06%) and 27 males (52.94%), with a mean group age of ~63 years (Table 1). Most participants were non-Hispanic whites (60.78%), reported an educational level higher than high school (58.82%), and were non-smokers (54.90%). However, only one statistically significant association was found between the self-report of periodontitis and sociodemographic data, namely the marital status (*p* = 0.025). The “no periodontitis” group presented a higher number of singles (28.57%), and the “periodontitis” group presented the majority of married/living with partner status (65.22%).

**Table 1:** General characteristics of PD patients according to the self-report of periodontitis.

Variables	Self-reported		*p*-value	Overall (*n* = 51)
	Without Periodontitis (*n* = 28)	Periodontitis (*n* = 23)		
**Age (years), mean (SD)**	65.36 (14.79)	60.04 (14.39)	0.133	62.96 (14.71)
**Females,** ***n*** **(%)**	14 (50.0)	10 (43.48)	0.855	24 (47.06)
**Ethnicity,** ***n*** **(%)**				
Mexican American	1 (3.57)	1 (4.35)	0.352	2 (3.92)
Other Hispanic	1 (3.57)	4 (17.39)		5 (9.80)
Non-Hispanic white	17 (60.71)	14 (60.87)		31 (60.78)
Non-Hispanic black	7 (25.00)	2 (8.70)		9 (17.65)
Other Race—including multi-racial	2 (7.14)	2 (8.70)		4 (7.84)
**Educational Level,** ***n*** **(%)**				
<High school	8 (28.57)	5 (21.74)	0.852	13 (25.49)
High school	5 (17.86)	3 (13.04)		8 (15.69)
>High school	15 (53.57)	15 (65.22)		30 (58.82)
**Marital Status,** ***n*** **(%)**				
Single	8 (28.57)	3 (13.04)	**0.025**	11 (21.57)
Married/Living with partner	14 (50.00)	15 (65.22)		29 (56.86)
Divorced/Separated/ Widowed	6 (21.43)	5 (21.74)		11 (21.57)
**FI/PR, mean (SD)**	2.62 (1.71)	1.92 (1.46)	0.221	2.31 (2.08)
**Smoking status,** ***n*** **(%)**				
Non-smokers	14 (50.00)	14 (60.87)	0.552	28 (54.90)
Former smokers	4 (14.29)	4 (17.39)		8 (15.69)
Active smokers	10 (35.71)	5 (21.74)		15 (29.41)
**Chronic medical conditions, mean (SD)**	2.75 (2.25)	5.91 (1.87)	0.406	2.55 (2.08)
**Diabetes,** ***n*** **(%)**	5 (17.86)	8 (34.78)	0.279	13 (25.49)
**Hba1c, mean (SD)**	6.03 (1.00)	5.91 (1.24)	0.064	5.98 (1.10)
**Hypertension,** ***n*** **(%)**	19 (67.86)	15 (65.22)	1.000	34 (66.67)
**SBP, mean (SD)**	141.95 (25.29)	129.86 (24.07)	0.173	136.50 (25.24)
**DBP, mean (SD)**	77.36 (12.39)	70.15 (8.88)	0.176	74.11 (11.43)
**Missing teeth, mean (SD)**	6.82 (6.60)	8.26 (6.66)	0.537	7.47 (6.60)

*DBP* Diastolic Blood Pressure, *FI/PR* Family income/poverty ratio, *Hba1c* Hemoglobin A1C level, *n* number of cases, *SBP* Systolic Blood Pressure, *SD* Standard Deviation.

*Mann–Whitney test for continuous variables and Chi-square test for categorical variables.

Regarding general health status, this group of PD patients presented a mean value of 2.55 total chronic medical conditions, 25.49% suffered from diabetes mellitus, and 66.67% from hypertension, although cases were evenly distributed (Table 1). Also, the sample presented approximately a mean value of 7.47 missing teeth. All in all, no statistically significant differences were found on general health covariates according to the self-report of periodontitis.

**Table 2:** Hematologic and biochemical levels of PD patients according to the self-report of periodontitis.

Variables	Self-reported		*p*-value*	Overall (*n* = 51)
	Without periodontitis (*n* = 28)	Periodontitis (*n* = 23)		
**Biochemical parameters, mean (SD)**				
hs-CRP (mg/L)	1.99 (2.03)	5.36 (6.37)	**0.031**	3.51 (4.82)
Total Cholesterol (mmol/L)	4.83 (0.82)	4.58 (1.17)	0.389	4.71 (1.01)
HDL-Cholesterol (mmol/L)	1.56 (0.56)	1.35 (0.39)	0.515	1.46 (0.50)
**Hematologic parameters, mean (SD)**				
WBC count (10^9^/L)	6.88 (2.23)	7.89 (2.24)	0.103	7.33 (2.31)
Lymphocyte percent (%)	29.35 (11.83)	28.03 (8.75)	**0.007**	28.76 (10.68)
Monocyte percent (%)	8.81 (2.64)	8.31 (2)	0.850	8.58 (2.41)
Segmented neutrophils percent (%)	58.16 (12.21)	60.46 (9.05)	0.596	59.20 (11.07)
Eosinophils percent (%)	2.87 (1.88)	2.44 (1.21)	0.513	2.68 (1.64)
Basophils percent (%)	0.95 (0.4)	0.87 (0.29)	0.682	0.91 (0.36)
Lymphocytes (10^9^/L)	1.93 (0.77)	2.17 (0.76)	0.214	2.04 (0.78)
Monocytes (10^9^/L)	0.57 (0.19)	0.64 (0.2)	0.946	0.60 (0.20)
Segmented neutrophils (10^9^/L)	4.12 (1.86)	4.83 (1.77)	0.142	4.44 (1.87)
Eosinophils (10^9^/L)	0.2 (0.15)	0.2 (0.11)	0.696	0.20 (0.13)
Basophils (10^9^/L)	0.07 (0.05)	0.07 (0.05)	0.886	0.07 (0.05)
RBC count (10^12^/L)	4.7 (0.4)	4.65 (0.59)	0.643	4.68 (0.50)
Hemoglobin (g/dL)	13.91 (1.42)	13.5 (1.67)	0.872	13.73 (1.57)
Hematocrit (%)	41.92 (3.77)	40.59 (4.42)	0.344	41.32 (4.17)
Mean cell volume (fL)	89.29 (5.11)	88.01 (9.34)	0.400	88.71 (7.43)
Mean cell hemoglobin (pg)	29.62 (2.29)	29.28 (3.76)	0.985	29.47 (3.07)
Mean Cell Hgb Conc. (g/dL)	33.15 (0.95)	33.2 (1.28)	0.519	33.17 (1.13)
RDC width (%)	14.28 (1.06)	15.08 (1.55)	0.052	14.64 (1.37)
Platelet count (1000 cells/uL)	248.46 (87.7)	219.04 (77.86)	0.195	235.20 (85.52)
MPV (fL)	8.22 (0.77)	8.33 (0.86)	0.726	8.27 (0.82)

^*^ Mann–Whitney test.

*WBC* White Blood Cells, *RBC* Red Blood Cells, *MCV* Mean Cell Volume, *MCH* Mean Cell Hemoglobin, *MCHC* Mean Cell Hemoglobin Concentration, *RCD* Red Cell Distribution, *MPV* Mean Platelet Volume.

### Blood and biochemical parameters

The levels of biochemical parameters and complete blood count with 5-part differential were analyzed in order to assess the systemic status of these participants according to the self-reported periodontal status (Table 2).

Overall, statistically significant differences were found for hs-CRP levels (*p* = 0.031) and lymphocyte percentage (*p* = 0.007). The “periodontitis” group presented higher mean levels of hs-CRP (5.36 vs. 1.99 mg/L) when compared to the “without periodontitis” group, while the “without periodontitis” group presented a higher mean percentage of lymphocytes when compared to the “periodontitis” group (29.35 vs. 28.03%).

In order to explore potential confounding variables on the hs-CRP values, we observed that PD patients with diabetes mellitus (*p* = 0.130), hypertension (*p* = 0.844), coronary heart disease (*p* = 0.405), emphysema (*p* = 0.365), asthma (*p* = 0.184), hepatic conditions (*p* = 0.888) or cancer (*p* = 0.354) had non-significant differences in serum levels of this marker. Similarly, active smokers did not present significant differences in serum levels of hs-CRP (*p* = 0.343).

## Discussion

The results of the present study showed that self-reported periodontitis is associated with higher circulating levels of hs-CRP in PD patients. No differences were found on white cells, red cells, and platelets according to the self-reported periodontal status of PD individuals, except for the lymphocyte percentage.

With this study, we ultimately aimed to strengthen the existing hypothesis that PD patients with periodontitis carry a systemic inflammatory burden, which can be translated into higher hs-CRP levels^27^. In fact, hs-CRP is one of the circulatory inflammatory markers previously known to be aggravated in patients with the periodontal disease when compared to healthy control groups^30–32^. Therefore, our results align and corroborate the previous lines of evidence in this regard^32^. Furthermore, to explore the hypothesis that the concomitant presence of other systemic conditions may interfere with the high hs-CRP levels in PD patients, we compared hs-CRP levels in PD patients with and without diabetes mellitus (*p* = 0.130), hypertension (*p* = 0.844), coronary heart disease (*p* = 0.405), emphysema (*p* = 0.365), asthma (*p* = 0.184), hepatic conditions (*p* = 0.888) or cancer (*p* = 0.354). No statistically significant differences were found, which indicates that the presence of other systemic diseases was not a confounding factor to the elevated hs-CRP levels in PD patients. Likewise, an active-smoker status was also not a confounding factor to high serum levels of hs-CRP (*p* = 0.343).

The clinical relevance of elevated CRP is worth discussing. This serum biomarker is mostly produced hepatically, triggered by acute and/or chronic inflammatory events^29^. In the past years, CRP has been shown to play a key role in the management of inflammatory diseases such as cardiovascular diseases^33^, neurodegenerative diseases (such as Alzheimer’s Disease (AD) and PD)^28,34^ or even periodontitis^27^. In addition to activating the complement classical pathway, CRP also binds to several tissues and membranes propelling the inflammatory reaction through cytokines and nuclear antigens. In what PD concerns, this may be of importance because systemic and cerebral inflammation is increasingly cited in its pathophysiological basis regarded as a syndrome by many^35^. Besides, neuroinflammation has been shown to be an important contributor to the pathogenesis of the Parkinsonian process and may aggravate the process of nigral neurodegeneration in animal models of PD^36^. Furthermore, in the periodontitis-PD link, and besides the established effects PD impairments cause in oral health that may ultimately lead to the development of periodontitis^9–16,24^, the infectious nature of periodontitis may have implications on gut microbiota^37^ which is known to be abnormal in PD^36^.

Self-reported periodontitis is a validated, efficient, and accepted measure of periodontitis cases, with higher validity upon a combination of several self-report questions^20–22^. In fact, the self-report strategy has been previously validated in other contexts, such as to identify cases of hypertension, diabetes mellitus, hypercholesterolemia^38^, risk factors for cardiovascular disease^39^, and even bruxism in PD patients^40^. All in all, self-report enables larger scale epidemiologic studies and low-cost surveillance of symptoms, risk factors, and diseases of interest^20^.

However, even though the periodontitis-leukocytosis link is well established—especially given the infectious nature of periodontitis whose effects summons WBC to the lesioned site^26,41,42^—the self-reported “periodontitis” group presented slightly lower lymphocyte percentage when compared to the “without periodontitis” group. This can be explained through the fact that elevated levels of WBC would be more probable upon clinical diagnosis of periodontitis cases. Therefore, as the clinical periodontal diagnosis is far more preferable and reliable, the use of self-reported measures of periodontitis stands as a limitation in this study, even though this method has been previously validated^20–22^ and provides a cost-effective means of great-scale monitoring of oral health^20^. Furthermore, disease severity and activity could not be appraised through a self-report method, which is also fully reliable on the patient’s knowledge of the disease and full awareness of a previous clinical diagnosis. Thus, the possibility of unmeasured confounding through this method of identification of periodontitis cases cannot be discarded^23^.

Additionally, the sample size may be considered limited and thus a shortcoming of this study. Perhaps, this might be because we are trying to signpost an underappreciated condition of difficult diagnosis^5^. Despite the small sample number, the collected data is of clinical significance, and we hope to pave the way for future larger studies on this condition worldwide (for instance using the MDS non-motor study group network). Also, the secondary study design based on the available NHANES data has been previously applied and accepted in several recent studies, including the PD-case selection method employed^26,43^. Nonetheless, due to the used PD-case selection method, data on disease duration was not available, which would have been relevant to evaluate disease staging. Furthermore, the observational nature of the study impairs the conclusion of causality, thus robust evidence has been reported regarding periodontitis increasing circulating levels of CRP and hs-CRP^42^.

All in all, the present study evaluates the association between self-reported periodontitis and hs-CRP levels in PD patients. Hence, future research should continue to focus on the systemic repercussions of the periodontitis infection in PD patients, in the hopes of potentially clarifying the causality of the PD-periodontitis link. Furthermore, future research including in-depth clinical measures of periodontitis (such as periodontal pocket depth and clinical attachment loss), will provide further confirmation on the association with circulating systemic inflammatory surrogates.

## Methods

### Study design

In this secondary study, data was extracted and further analyzed from the National Health and Nutrition Examination Survey (NHANES), a representative and stratified multistage health-related survey conducted on non-institutionalized U.S. citizens. The STrengthening the Reporting of OBservational studies in Epidemiology (STROBE) guideline was followed (Table S1, Supplementary Materials)^44^.

### Setting, participants, and study size

Data from the NHANES 2015–2016 and 2017–2018 databases were used for the present study. Our analysis deemed the following inclusion criteria: 18 years of age or older; and undertaking secure PD medication regimens. Edentulous patients, missing data (on sociodemographic and/or systemic health information), and unsecure PD medication regimens as previously defined (Cabergoline, Orphenadrine, and Pramipexole)^26^ were excluded.

Detailed information on sampling, design, and medical records are displayed at www.cdc.gov/nchs/nhanes.htm (accessed in April 2021). Health-related data-collection protocols from the NHANES 2015–2016 and 2017–2018 datasets underwent revision and approval by the Centers for Disease Control (CDC) and Prevention National Increase for Health Statistics Research (NCHS) Ethics Review Board, Atlanta USA, and all study participants provided written informed consent^45^.

### Variables and data measurement

#### PD definition

PD cases were identified in the NHANES database through the report of specific PD medications, according to a previous study^26^. Hence, the reported use of Benztropine, Carbidopa, Levodopa, Ropinirole, Methyldopa, Entacapone, and Amantadine were considered PD medications indicative of PD, therefore validating a PD case^46,47^. Cabergoline, Orphenadrine, and Pramipexole all present other known clinical applications apart from PD-Cabergoline is used to treat high levels of prolactin hormone^48^, Orphenadrine is used to treat muscle spasms in musculoskeletal conditions^49^ and Pramipexole is also used to treat restless legs syndrome (RLS)^50^—and therefore were considered unsecure medications for the selection of PD cases.

#### Periodontitis definition

Periodontitis cases were pinpointed through a positive self-report on either one of the following oral health-related (OHR) questions, all regarding the moment when the survey was applied: “Do you think you might have gum disease?”, “Ever had treatment for gum disease?” and “Ever been told of bone loss around teeth?”. This method of self-reporting periodontitis has been previously validated and is indicative of a periodontitis case^20–22^.

#### Demographic characteristics

Age, gender, ethnicity, level of education, marital status, family income to poverty ratio, and smoking status were the self-reported sociodemographic variables collected and analyzed from NHANES datasets.

Regarding ethnicity, “Mexican American”, “Other Hispanic”, “Non-Hispanic White”, “Non-Hispanic Black” and “Other race—including multi-racial” were the used designations, as indicated in the NHANES self-reported questionnaires^43^.

The level of education in individuals aged over 20 was categorized as follows: “<high school” (including <9th grade, 9–11th grade, and 12th grade with no diploma), “high school” (including high school grad/GED or equivalent) and “>high school” (including some college or AA degree and college graduate or above)^51^.

Concerning patients’ marital status, the used definition included “single” (never married), “married/living with a partner”, and “divorced/separated/widowed”^52^.

With regards to the reported family income to poverty ratio, a continuous score from 0 to 5 was given: “0” corresponding to no income, “5” corresponding to an income 5 or more times above the federal poverty threshold^53^.

At last, smoking status was defined as “active smokers” (reporting a consumption of ≥100 cigarettes during their lifetime and still currently smoking), “former smokers” (reporting smoking ≥100 cigarettes during their lifetime and presently ceased smoking) and non-smokers (reporting having smoked <100 cigarettes during their lifetimes)^26^.

#### Health characteristics

The systemic health status of the included participants was overall characterized through a sum of chronic medical conditions—asthma, psoriasis, gout, congestive heart failure, coronary heart disease, angina, heart attack, stroke, emphysema, thyroid, bronchitis, liver, and cancer—which was statistically considered a continuous variable. Furthermore, Diabetes Mellitus (DM) was separately defined through self-report information and confirmed with glycated hemoglobin levels (Hba1c)^54^. Levels of Hba1c > 8% were considered uncontrolled DM cases^55^. Also, high blood pressure cases were defined from previous self-reports of medical-informed hypertension and were further confirmed with systolic and diastolic blood pressure levels (>140 mmHg and >90 mmHg, respectively)^56^.

#### Blood and biochemical parameters

Serum fractions of hs-CRP (mg/L), HDL-cholesterol (mg/dL), and total cholesterol were analyzed from blood specimens of the NHANES database^57,58^.

Also, complete blood count with 5-part differential data was gathered, and information on white Blood Cell (WBC) count (10^9^/L), percentage of Lymphocyte (%), percentage of Monocyte (%), percentage of Segmented Neutrophils (%), percentage of Eosinophils (%), percentage of Basophils (%), Lymphocyte (10^9^/L), Monocyte (10^9^/L), Segmented neutrophils (10^9^/L), Eosinophils (10^9^/L), Basophils (10^9^/L), Red Blood Cell (RBC) count (10^12^/L), Hemoglobin (g/dL), Hematocrit (%), Mean Cell Volume (MCV) (fL), Mean Cell Hemoglobin (MCH) (pg), Mean Cell Hemoglobin Concentration (MCHC) (g/dL), Red Cell Distribution (RCD) width (%), Platelet count (1000 cells/uL) and Mean Platelet Volume (MPV) (fL) was analyzed.

Blood collection occurred ~3 weeks following interviews, as detailed in https://wwwn.cdc.gov/nchs/nhanes/continuousnhanes/manuals.aspx?BeginYear=2015 (accessed in April 2021).

### Data management, analysis, and statistical methods

Data analysis of the 2015–2016 and 2017–2018 NHANES datasets was conducted through IBM SPSS Statistics version 26.0.0.0 for Macintosh (Armonk, New York, IBM Corp.). Data were uploaded via SAS Universal Viewer and handled with Microsoft Excel. Continuous variables are reported through mean ± standard deviation (SD), while the number of cases (*n*) and percentage (%) represent categorical variables distribution among group categories. Upon assessment of data non-normality and homoscedasticity, Mann–Whitney test was applied for comparison of continuous variables. Chi-square test was used to evaluate association between the categorical variables. A 5% significance level was used in all inferential analyses.

### Reporting Summary

Further information on research design is available in the Nature Research Reporting Summary linked to this article.

## Supplementary information

Final Supplementary Materials

Reporting Summary Checklist

## Data Availability

The analyzed data that supports the findings of this study are available in the NHANES database, a publicly accessible repository that does not issue DOIs, www.cdc.gov/nchs/nhanes.htm. The used datasets can be located under the “Survey Data and Documentation” tab, followed by the “NHANES 2015–2016” and “NHANES 2017–2018” databases tabs. “Demographics Data”, “Examination Data”, “Laboratory Data” and “Questionnaire Data” were consulted.
