# Supplementary material for: Self-reported periodontitis and C-reactive protein in Parkinson’s disease: a cross-sectional study of two American cohorts
Source: NPJ Parkinsons Dis. 2022 Apr 13;8:40. doi: 10.1038/s41531-022-00302-1 (PMC9008053; doi:10.1038/s41531-022-00302-1)
Supplement: Supplementary file 2 — Reporting Summary Checklist [file 41531_2022_302_MOESM2_ESM.pdf]

nature portfolio

Corresponding author(s): Patrícia Lyra

Last updated by author(s): Mar 7, 2022

Reporting Summary

Nature Portfolio wishes to improve the reproducibility of the work that we publish. This form provides structure for consistency and transparency in reporting. For further information on Nature Portfolio policies, see our Editorial Policies and the Editorial Policy Checklist.

Statistics

For all statistical analyses, confirm that the following items are present in the figure legend, table legend, main text, or Methods section.

n/a	Confirmed	
☐	☒	The exact sample size (n) for each experimental group/condition, given as a discrete number and unit of measurement
☐	☒	A statement on whether measurements were taken from distinct samples or whether the same sample was measured repeatedly
☐	☒	The statistical test(s) used AND whether they are one- or two-sided<br>*Only common tests should be described solely by name; describe more complex techniques in the Methods section.*
☐	☒	A description of all covariates tested
☒	☐	A description of any assumptions or corrections, such as tests of normality and adjustment for multiple comparisons
☐	☒	A full description of the statistical parameters including central tendency (e.g. means) or other basic estimates (e.g. regression coefficient) AND variation (e.g. standard deviation) or associated estimates of uncertainty (e.g. confidence intervals)
☐	☒	For null hypothesis testing, the test statistic (e.g. F, t, r) with confidence intervals, effect sizes, degrees of freedom and P value noted<br>*Give P values as exact values whenever suitable.*
☒	☐	For Bayesian analysis, information on the choice of priors and Markov chain Monte Carlo settings
☒	☐	For hierarchical and complex designs, identification of the appropriate level for tests and full reporting of outcomes
☒	☐	Estimates of effect sizes (e.g. Cohen's d, Pearson's r), indicating how they were calculated

Our web collection on statistics for biologists contains articles on many of the points above.

Software and code

Policy information about availability of computer code

Data collection	In this secondary study, data was extracted and further analyzed from the National Health and Nutrition Examination Survey (NHANES), a representative and stratified multistage health-related survey conducted on non-institutionalized U.S. citizens. Detailed information on sampling, design and medical records are displayed at www.cdc.gov/nchs/nhanes.htm (accessed in April 2021).
Data analysis	Data analysis of the 2015-2016 and 2017-2018 NHANES datasets was conducted through IBM SPSS Statistics version 26.0.0.0 for Macintosh (Armonk, New York, IBM Corp.). Data were uploaded via SAS Universal Viewer and handled with Microsoft Excel. Continuous variables are reported through mean ± standard deviation (SD), while the number of cases (n) and percentage (%) represent categorical variables distribution among group categories. Upon assessment of data non-normality and homoscedasticity, Mann-Whitney test was applied for comparison of continuous variables. Chi-square test was used to evaluate association between the categorical variables. A 5% significance level was used in all inferential analyses.

For manuscripts utilizing custom algorithms or software that are central to the research but not yet described in published literature, software must be made available to editors and reviewers. We strongly encourage code deposition in a community repository (e.g. GitHub). See the Nature Portfolio guidelines for submitting code & software for further information.

Data

Policy information about availability of data

All manuscripts must include a data availability statement. This statement should provide the following information, where applicable:

- Accession codes, unique identifiers, or web links for publicly available datasets
- A description of any restrictions on data availability
- For clinical datasets or third party data, please ensure that the statement adheres to our policy

The data analyzed in this study is available in a publicly accessible repository that does not issue DOIs, www.cdc.gov/nchs/nhanes.htm.

Field-specific reporting

Please select the one below that is the best fit for your research. If you are not sure, read the appropriate sections before making your selection.

☒ Life sciences ☐ Behavioural & social sciences ☐ Ecological, evolutionary & environmental sciences

For a reference copy of the document with all sections, see nature.com/documents/nr-reporting-summary-flat.pdf

Life sciences study design

All studies must disclose on these points even when the disclosure is negative.

Sample size	Our analysis deemed the following inclusion criteria: 18 years of age or older; and undertaking secure PD medication regimens. The collected data is of clinical significance, and we hope to pave the way for future larger studies on this condition worldwide.
Data exclusions	Edentulous patients, missing data (on sociodemographic and/or systemic health information) and unsecure PD medication regimens as previously defined (Cabergoline, Orphenadrine and Pramipexole) were excluded.
Replication	All measures were described thoroughly to allow future replicability.
Randomization	N/A
Blinding	N/A

Reporting for specific materials, systems and methods

We require information from authors about some types of materials, experimental systems and methods used in many studies. Here, indicate whether each material, system or method listed is relevant to your study. If you are not sure if a list item applies to your research, read the appropriate section before selecting a response.

Materials & experimental systems

n/a	Involved in the study	
☒	☐	Antibodies
☒	☐	Eukaryotic cell lines
☒	☐	Palaeontology and archaeology
☒	☐	Animals and other organisms
☐	☒	Human research participants
☐	☒	Clinical data
☒	☐	Dual use research of concern

Methods

n/a	Involved in the study	
☒	☐	ChIP-seq
☒	☐	Flow cytometry
☒	☐	MRI-based neuroimaging

Human research participants

Policy information about studies involving human research participants

Population characteristics	The sample consisted of 24 females (47.06%) and 27 males (52.94%), with the mean group age approximately of 63 years (Table 1). Most participants were non-hispanic white (60.78%), reported an educational level higher than high school (58.82%) and were non-smokers (54.90%). However, only one statistically significant association was found between the self-report of periodontitis and sociodemographic data, namely the marital status (p = 0.025). The "no periodontitis" group presented a higher number of singles (28.57%), and the "periodontitis" group presented the majority of married/living with partner status (65.22%).
Recruitment	In this secondary study, data was extracted and further analyzed from the National Health and Nutrition Examination Survey (NHANES), a representative and stratified multistage health-related survey conducted on non-institutionalized U.S. citizens.

	Detailed information on sampling, design and medical records are displayed at www.cdc.gov/nchs/nhanes.htm (accessed in April 2021).
Ethics oversight	Health-related data-collection protocols from the NHANES 2015-2016 and 2017-2018 datasets underwent revision and approval by the Centers for Disease Control (CDC) and Prevention National Increase for Health Statistics Research (NCHS) Ethics Review Board, Atlanta USA, and all study participants provided written informed consent.

Note that full information on the approval of the study protocol must also be provided in the manuscript.

Clinical data

Policy information about clinical studies

All manuscripts should comply with the ICMJE guidelines for publication of clinical research and a completed CONSORT checklist must be included with all submissions.

Clinical trial registration	N/A
Study protocol	N/A
Data collection	N/A
Outcomes	N/A
